# A fast continuous time approach with time scaling for nonsmooth convex optimization

**DOI:** 10.1186/s13662-022-03744-2

**Published:** 2022-12-16

**Authors:** Radu Ioan Boţ, Mikhail A. Karapetyants

**Affiliations:** Faculty of Mathematics, University of Vienna, Oskar-Morgenstern-Platz 1, 1090 Vienna, Austria

**Keywords:** 37N40, 46N10, 49M99, 65K05, 65K10, 90C25, Nonsmooth convex optimization, Damped inertial dynamics, Hessian-driven damping, Time scaling, Moreau envelope, Proximal operator

## Abstract

In a Hilbert setting, we study the convergence properties of the second order in time dynamical system combining viscous and Hessian-driven damping with time scaling in relation to the minimization of a nonsmooth and convex function. The system is formulated in terms of the gradient of the Moreau envelope of the objective function with a time-dependent parameter. We show fast convergence rates for the Moreau envelope, its gradient along the trajectory, and also for the system velocity. From here, we derive fast convergence rates for the objective function along a path which is the image of the trajectory of the system through the proximal operator of the first. Moreover, we prove the weak convergence of the trajectory of the system to a global minimizer of the objective function. Finally, we provide multiple numerical examples illustrating the theoretical results.

## Introduction

Let *H* be a real Hilbert space endowed with the scalar product $\langle \cdot , \cdot \rangle $ and norm $\| x \| = \sqrt{\langle x, x \rangle}$ for $x \in H$. In connection with the minimization problem

$$ \min_{x \in H} \Phi (x), $$

we will study the asymptotic behavior of the second order in time evolution equation

1 $$ \ddot{x}(t) + \frac{\alpha}{t} \dot{x}(t) + \beta (t) \frac{d}{dt} \nabla \Phi _{\lambda (t)}\bigl(x(t)\bigr) + b(t) \nabla \Phi _{\lambda (t)}\bigl(x(t)\bigr) = 0, $$

with initial conditions $x(t_{0}) = x_{0} \in H$, $\dot{x}(t_{0}) = u_{0} \in H$, where $\alpha \geq 1 $, $t_{0} >0$, and $\beta : [t_{0}, +\infty ) \longrightarrow [0, +\infty )$ and $b, \lambda : [t_{0}, +\infty ) \longrightarrow (0, +\infty ) $ are differentiable functions.

We assume that $\Phi : H \longrightarrow \overline{\mathbb{R}} = \mathbb{R} \cup \{ \pm \infty \} $ is a proper, convex and lower semicontinuous function and denote by $\Phi _{\lambda}: H \longrightarrow \mathbb{R} $ its Moreau envelope of parameter $\lambda > 0$. In addition, we assume that argminΦ, the set of global minimizers of Φ, is not empty and denote by $\Phi ^{*}$ the optimal objective value of Φ.

Our aim is to derive rates of convergence for the Moreau envelope of the objective function and the objective function itself to $\Phi ^{*}$, as well as for the gradient of the Moreau envelope of the objective function and the velocity of the trajectory to zero in terms of the Moreau parameter function *λ* and the time scaling function *b*. In addition, we will provide a setting that also guarantees the weak convergence of the trajectory of the dynamical system to a minimizer of Φ. The theoretical results will be illustrated by multiple numerical experiments.

### Historical remarks

Inertial dynamics were introduced by Polyak in [23] in the form of the so-called heavy ball with friction method

$$ \ddot{x}(t) + \alpha \dot{x}(t) + \nabla \Phi \bigl(x(t)\bigr) = 0, $$

with fixed viscous coefficient $\alpha >0$, to accelerate the gradient method for the minimization of a continuous differentiable function $\Phi : H \to \mathbb{R}$. This system was later studied by Alvarez–Attouch [3, 4] and by Attouch–Goudou–Redont [11]. In these works, for a convex function Φ, an asymptotic convergence rate of $\Phi (x(t))$ to $\Phi ^{*}$ of order $O ( \frac{1}{t} )$ as $t \to +\infty $, as well as an improvement for a strongly convex function Φ to an exponential rate of convergence, was proved. The weak convergence of the trajectories to a minimizer of Φ was also established.

A major step to obtain faster asymptotic convergence in the convex regime was done by Su–Boyd–Candes [24], by considering in the second order dynamical system an asymptotic vanishing damping coefficient

2 $$ \ddot{x}(t) + \frac{\alpha}{t} \dot{x}(t) + \nabla \Phi \bigl(x(t)\bigr) = 0, $$

for $t \geq t_{0}$ and $\alpha \geq 3$. Second order dynamical systems with variable and vanishing damping coefficients for optimization were studied, for instance, in [17–19]. The system (2) corresponds to a continuous version of Nesterov’s accelerated gradient method [21]. For the function values, rates of convergence of

$$ \Phi \bigl(x(t)\bigr) - \Phi ^{*} = O \biggl( \frac{1}{t^{2}} \biggr)\quad \text{as } t \to +\infty $$

were obtained. For $\alpha >3$, in [9], it was shown that the trajectory of (2) converges weakly to an element of argminΦ, and in [13, 20], the asymptotic convergence rate of the function values was improved to $o ( \frac{1}{t^{2}} )$ as $t \to +\infty $.

The following system, combining asymptotic vanishing damping with the Hessian-driven damping, was proposed by Attouch–Peypouquet–Redont in [15]

3 $$ \ddot{x}(t) + \frac{\alpha}{t} \dot{x}(t) + \beta \nabla ^{2} \Phi \bigl(x(t)\bigr) \dot{x}(t) + \nabla \Phi \bigl(x(t)\bigr) = 0 $$

for $t \geq t_{0} $, where $\Phi : H \longrightarrow \mathbb{R}$ twice continuously differentiable and convex, $\alpha \geq 3$ and $\beta \geq 0$. The Hessian-driven damping has a natural link with Newton’s method and gives rise to dynamical inertial Newton systems [1]. The system (3) preserves the convergence properties of (2), while having for $\beta >0$ other important features, namely,

$$ \lim_{t \rightarrow +\infty} \bigl\Vert \nabla \Phi \bigl(x(t)\bigr) \bigr\Vert =0 \quad \text{and}\quad \int _{t_{0}}^{+\infty} t^{2} \bigl\Vert \nabla \Phi \bigl(x(t)\bigr) \bigr\Vert ^{2}\,dt < + \infty .$$

In addition, possible oscillations exhibited by the solutions of (2) are neutralized by (3).

### Time scaling

Time scaling of the dynamical system (2) was used to accelerate the rate of convergence of the values of the function Φ along the trajectory. The system (2) becomes through time scaling a dynamical system of the form

4 $$ \ddot{x}(t) + \frac{\alpha}{t} \dot{x}(t) + b(t) \nabla \Phi \bigl(x(t)\bigr) = 0, $$

where $\alpha \geq 3$, and $b : [t_{0}, +\infty ) \longrightarrow (0, +\infty )$ is a continuous scalar function, as it was introduced and studied by Attouch–Chbani–Riahi in [10]. For (4), it was shown that

$$ \Phi \bigl(x(t)\bigr) - \Phi ^{*} = O \biggl( \frac{1}{t^{2} b(t)} \biggr) \quad \text{as } t \to +\infty , $$

and a convergence rate can be improved to $o ( \frac{1}{t^{2}b(t)} )$ as $t \to +\infty $, if $\alpha >3$.

In [7, 8] (see also [5]), the dynamical system

5 $$ \ddot{x}(t) + \frac{\alpha}{t} \dot{x}(t) + \beta (t) \nabla ^{2} \Phi \bigl(x(t)\bigr) \dot{x}(t) + b(t) \nabla \Phi \bigl(x(t) \bigr) = 0, $$

which combines viscous and Hessian-driven damping with time scaling, where $\alpha \geq 1$ and $\beta , b : [t_{0}, +\infty ) \longrightarrow (0, +\infty )$ are functions with appropriate differentiability properties, was investigated. A quite general setting formulated in terms of the dynamical system parameter functions was identified in which the properties of (5) concerning the convergence of the function values are preserved, while the gradient of Φ strongly converges along the trajectory to zero, and the trajectory converges weakly to a minimizer of the objective function. In [7, 8], a numerical algorithm obtained via time discretization of (5) was studied, exhibiting analogous convergence properties to the dynamical system.

### Nonsmooth optimization

The Moreau envelope of a proper, convex and lower semicontinuous function $\Phi : H \to \overline{\mathbb{R}}$ has played a significant role in the literature when designing continuous-time approaches and numerical algorithms for the minimization of Φ. This is defined as

$$ \Phi _{\lambda}: H \to \mathbb{R}, \qquad \Phi _{\lambda} (x) = \inf_{y \in H} \biggl\{ \Phi (y) + \frac{1}{2 \lambda} \Vert x - y \Vert ^{2} \biggr\} , $$

where $\lambda > 0$ is called the parameter of the Moreau envelope (see, for instance, [16]). For every $\lambda >0$, the functions Φ and $\Phi _{\lambda}$ share the same optimal objective value and the same set of minimizers. In addition, $\Phi _{\lambda}$ is convex and continuously differentiable with

6 $$\begin{aligned} \nabla \Phi _{\lambda} (x) = \frac{1}{\lambda} \bigl( x - \operatorname{prox}_{ \lambda \Phi} (x)\bigr) \quad \forall x \in H, \end{aligned}$$

and $\nabla \Phi _{\lambda}$ is $\frac{1}{\lambda}$-Lipschitz continuous. Here,

$$ \operatorname{prox}_{\lambda \Phi}: H \to H, \qquad \operatorname{prox}_{ \lambda \Phi} (x) = \operatorname*{argmin}_{y \in H} \biggl\{ \Phi (y) + \frac{1}{2 \lambda} \Vert x - y \Vert ^{2} \biggr\} $$

denotes the proximal operator of Φ of parameter *λ*. For every $x \in H$ and $\lambda , \mu >0$, we have

7 $$ \bigl\Vert \operatorname{prox}_{\lambda \Phi}(x) - \operatorname{prox}_{\mu \Phi}(x) \bigr\Vert \leq \vert \lambda - \mu \vert \bigl\Vert \nabla \Phi _{\lambda} (x) \bigr\Vert . $$

On the other hand, for every $x \in H$, the function $\lambda \in (0, +\infty ) \to \Phi _{\lambda }(x)$ is nonincreasing and differentiable (see, for instance, [6, Lemma A.1]), namely,

$$ \frac{d}{d \lambda} \Phi _{\lambda} (x) = -\frac{1}{2} \bigl\Vert \nabla \Phi _{\lambda} (x) \bigr\Vert ^{2} \quad \forall \lambda >0. $$

Attouch–Cabot considered in [6] (see also [14] for a more general approach for monotone inclusions) in connection with the minimization of the proper, convex and lower semicontinuous function $\Phi : H \to \overline{\mathbb{R}}$ the following second order differential equation

8 $$ \ddot{x}(t) + \frac{\alpha}{t} \dot{x}(t) + \nabla \Phi _{\lambda (t)} \bigl(x(t)\bigr) = 0 $$

for $t \geq t_{0}$, where $\alpha \geq 1$, and $\lambda : [t_{0}, +\infty ) \longrightarrow (0, +\infty )$ is continuously differentiable and nondecreasing. Convergence rates for the values of the Moreau envelope, as well as for the velocity of the system, were obtained

$$ \Phi _{\lambda (t)}\bigl(x(t)\bigr) - \Phi ^{*} = o \biggl( \frac{1}{t^{2}} \biggr) \quad \text{and}\quad \bigl\Vert \dot{x}(t) \bigr\Vert = o \biggl( \frac{1}{t} \biggr) \quad \text{as } t \to +\infty , $$

from where convergence rates for the Φ along $x(t)$ were deduced

$$ \begin{aligned} &\Phi \bigl( \operatorname{prox}_{\lambda (t) \Phi} \bigl(x(t)\bigr) \bigr) - \Phi ^{*} = o \biggl( \frac{1}{t^{2}} \biggr) \quad \text{and} \\ &\bigl\Vert \operatorname{prox}_{ \lambda (t) \Phi} \bigl(x(t)\bigr) - x(t) \bigr\Vert = o \biggl( \frac{\sqrt{\lambda (t)}}{t} \biggr) \quad \text{as } t \to +\infty . \end{aligned} $$

In addition, the weak convergence of the trajectories $x(t)$ to a minimizer of Φ as $t \to +\infty $ was established.

Attouch–László considered in [12] in the same context the dynamical system

9 $$ \ddot{x}(t) + \frac{\alpha}{t} \dot{x}(t) + \beta \frac{d}{dt} \nabla \Phi _{\lambda (t)}\bigl(x(t)\bigr) + \nabla \Phi _{\lambda (t)}\bigl(x(t)\bigr) = 0, $$

where $\alpha > 1$ and $\beta > 0$, and the term $\frac{d}{dt} \nabla \Phi _{\lambda (t)}(x(t))$ is inspired by the Hessian-driven damping, and its existence is justified almost everywhere since the mapping $t \to \nabla \Phi _{\lambda (t)}(x(t))$ is locally absolutely continuous (see, for example, [12, Lemma 1]). It was shown that for $\lambda (t) = \lambda t^{2}$, where $\lambda >0$, the system (9) inherits all major convergence properties of (8), and, in addition, the following convergence rates for the gradient of the Moreau envelope of parameter $\lambda (t)$ and its time derivative along $x(t)$ were established

$$ \bigl\Vert \nabla \Phi _{\lambda (t)} \bigl(x(t)\bigr) \bigr\Vert = o \biggl( \frac{1}{t^{2}} \biggr) \quad \text{and} \quad \biggl\Vert \frac{d}{dt} \nabla \Phi _{\lambda (t)} \bigl(x(t)\bigr) \biggr\Vert = o \biggl( \frac{1}{t^{2}} \biggr) \quad \text{as } t \to + \infty . $$

### Our contribution

In this paper, we derive a setting formulated in terms of $\alpha \geq 1$ and the parameter functions *β*, *b* and *λ* of the dynamical system (1) associated with the minimization of the proper, convex and lower semicontinuous function $\Phi : H \to \overline{\mathbb{R}}$, which allow us to prove

- convergence rates for the Moreau envelope, its gradient, and the velocity of the trajectory
  $$ \begin{aligned} &\Phi _{\lambda (t)}\bigl(x(t)\bigr) - \Phi ^{*} = o \biggl( \frac{1}{t^{2} b(t)} \biggr), \qquad \bigl\Vert \nabla \Phi _{\lambda (t)} \bigl(x(t)\bigr) \bigr\Vert = o \biggl( \frac{1}{t \sqrt{b(t) \lambda (t)}} \biggr) \quad \text{and} \\ &\bigl\Vert \dot{x}(t) \bigr\Vert = o \biggl( \frac{1}{t} \biggr) \end{aligned} $$
  as $t \to +\infty $, respectively;
- convergence rates for the objective function
  $$ \begin{aligned} &\Phi \bigl( \operatorname{prox}_{\lambda (t) \Phi} \bigl(x(t)\bigr) \bigr) - \Phi ^{*} = o \biggl( \frac{1}{t^{2} b(t)} \biggr) \quad \text{and} \\ &\bigl\Vert \operatorname{prox}_{\lambda (t) \Phi} \bigl(x(t)\bigr) - x(t) \bigr\Vert = o \biggl( \frac{\sqrt{\lambda (t)}}{t \sqrt{b(t)}} \biggr) \end{aligned} $$
  as $t \to +\infty $;
- the weak convergence of the trajectory $x(t)$ to a minimizer of Φ as $t \to +\infty $.

In addition, we provide a particular formulation of the derived general setting for the case when the parameter functions are chosen to be polynomials and illustrate the influence of the latter on the convergence behavior of the dynamical system by multiple numerical experiments.

### Existence and uniqueness of strong global solution

This section is devoted to the topic of the existence and uniqueness of a strong global solution of the system of our interest. To this aim, we will rewrite (1) as a system of the first order in time equations in the product space $H \times H$.

First, we assume that $\beta : [t_{0}, +\infty ) \longrightarrow [0, +\infty )$ is twice continuously differentiable with $\beta (t) >0$ for every $t \geq t_{0}$. We integrate (1) from $t_{0}$ to *t* to obtain

$$\begin{aligned} &\dot{x}(t) + \beta (t) \nabla \Phi _{\lambda (t)} \bigl(x(t)\bigr) + \int _{t_{0}}^{t} \biggl( \frac{\alpha}{s} \dot{x}(s) + b(s) \nabla \Phi _{\lambda (s)} \bigl(x(s)\bigr) \biggr) \,ds - \int _{t_{0}}^{t} \nabla \Phi _{\lambda (s)} \bigl(x(s)\bigr) \dot{\beta}(s) \,ds \\ &\quad {}- \bigl( \dot{x}(t_{0}) + \beta (t_{0}) \nabla \Phi _{\lambda (t_{0})} \bigl(x(t_{0})\bigr) \bigr) = 0. \end{aligned}$$

We denote $z(t) := \int _{t_{0}}^{t} ( \frac{\alpha}{s} \dot{x}(s) + ( b(s) - \dot{\beta}(s) ) \nabla \Phi _{\lambda (s)} (x(s)) ) \,ds - ( u_{0} + \beta (t_{0}) \nabla \Phi _{\lambda (t_{0})} (x_{0})) )$ for every $t \geq t_{0}$. Since $\dot{z}(t) = \frac{\alpha}{t}\dot{x}(t) + ( b(t) - \dot{\beta}(t) ) \nabla \Phi _{\lambda (t)} (x(t))$, we notice that (1) is equivalent to

$$ \textstyle\begin{cases} \dot{x}(t) + \beta (t) \nabla \Phi _{\lambda (t)} (x(t)) + z(t) = 0, \\ \dot{z}(t) - \frac{\alpha}{t} \dot{x}(t) - ( b(t) - \dot{\beta}(t) ) \nabla \Phi _{\lambda (t)}(x(t)) = 0, \\ x(t_{0}) = x_{0},\qquad z(t_{0}) = - (u_{0} + \beta (t_{0}) \nabla \Phi _{\lambda (t_{0})} (x_{0}) ). \end{cases} $$

After multiplying the first line by $b(t) - \dot{\beta}(t)$ and the second one by $\beta (t)$ and then summing them, we get rid of the gradient of the Moreau envelope in the second equation

$$ \textstyle\begin{cases} \dot{x}(t) + \beta (t) \nabla \Phi _{\lambda (t)} (x(t)) + z(t) = 0, \\ \beta (t) \dot{z}(t) + ( b(t) - \dot{\beta}(t) - \frac{\alpha \beta (t)}{t} ) \dot{x}(t) + ( b(t) - \dot{\beta}(t) ) z(t) = 0, \\ x(t_{0}) = x_{0},\qquad z(t_{0}) = - (u_{0} + \beta (t_{0}) \nabla \Phi _{\lambda (t_{0})} (x_{0}) ). \end{cases} $$

We denote $y(t) = \beta (t) z(t) + ( b(t) - \dot{\beta}(t) - \frac{\alpha \beta (t)}{t} ) x(t)$, and after simplification, we obtain for the dynamical system the following equivalent formulation

$$ \textstyle\begin{cases} \dot{x}(t) + \beta (t) \nabla \Phi _{\lambda (t)} (x(t)) + ( \frac{\dot{\beta}(t) - b(t)}{\beta (t)} + \frac{\alpha}{t} ) x(t) + \frac{1}{\beta (t)} y(t) = 0, \\ \dot{y}(t) + ( \ddot{\beta}(t) + \frac{3 b(t) \dot{\beta}(t) - 2 \dot{\beta}^{2}(t) - b^{2}(t)}{\beta (t)} + \frac{\alpha}{t} ( b(t) - \dot{\beta}(t) - \frac{\beta (t)}{t} ) - \dot{b}(t) ) x(t) + \frac{b(t) - 2 \dot{\beta}(t)}{\beta (t)} y(t) = 0, \\ x(t_{0}) = x_{0},\qquad y(t_{0}) = -\beta (t_{0}) (u_{0} + \beta (t_{0}) \nabla \Phi _{\lambda (t_{0})} (x_{0}) ) + ( b(t_{0}) - \dot{\beta}(t_{0}) - \frac{\alpha \beta (t_{0})}{t_{0}} ) x_{0}. \end{cases} $$

In case $\beta (t) = 0$ for every $t \geq t_{0}$, (1) can be equivalently written as

$$ \textstyle\begin{cases} \dot{x}(t) - y(t) = 0, \\ \dot{y}(t) + \frac{\alpha}{t} y(t) + b(t) \nabla \Phi _{\lambda (t)}(x(t)) = 0, \\ x(t_{0}) = x_{0}, \qquad y(t_{0}) = u_{0}. \end{cases} $$

Based on the two reformulations of the dynamical system (1), we can formulate the following existence and uniqueness result, which is a consequence of the Cauchy–Lipschitz theorem for strong global solutions. The result can be proved in the lines of the proofs of Theorem 1 in [12] or of Theorem 1.1 in [15] with some small adjustments.

#### Theorem 1

*Suppose that* $\beta : [t_{0}, +\infty ) \longrightarrow [0, +\infty )$ *is twice continuously differentiable such that either* $\beta (t) >0$ *for every* $t \geq t_{0}$ *or* $\beta (t) = 0$ *for every* $t \geq t_{0}$, *and there exists* $\lambda _{0} > 0$ *such that* $\lambda (t) \geq \lambda _{0}$ *for all* $t \geq t_{0}$. *Then*, *for every* $(x_{0}, u_{0}) \in H \times H $, *there exists a unique strong global solution* $x: [t_{0}, +\infty ) \mapsto H$ *of the continuous dynamics* (1), *satisfying the Cauchy initial conditions* $x(t_{0}) = x_{0}$ *and* $\dot{x}(t_{0}) = u_{0}$.

## Energy function and rates of convergence for function values

In this section, we will define an energy function for the dynamical system (1) and investigate its dissipativity properties. These will play a crucial role in the derivation of rates of convergence for the Moreau envelope of Φ and the objective function itself.

To shorten the calculations, we introduce the auxiliary function (see also [7, 8])

$$ w: [t_{0}, +\infty ) \to \mathbb{R}, \qquad w(t) = b(t) - \dot{\beta}(t) -\frac{\beta (t)}{t}. $$

For $z \in \operatorname*{argmin}\Phi $ and

10 $$ 0 \leq c \leq \alpha - 1, $$

consider the energy function $E_{c} : [t_{0},+\infty ) \rightarrow [0,+\infty )$,

$$\begin{aligned} \begin{aligned} E_{c}(t) & = \bigl( t^{2} w(t) + (\alpha -1-c) t \beta (t) \bigr) \bigl( \Phi _{\lambda (t)} \bigl(x(t)\bigr) - \Phi ^{*} \bigr) \\ &\quad {} + \frac{1}{2} \bigl\Vert c\bigl(x(t) - z\bigr) + t \dot{x}(t) + t \beta (t) \nabla \Phi _{\lambda (t)} \bigl(x(t)\bigr) \bigr\Vert ^{2} \\ &\quad{} + \frac{c(\alpha - 1 - c)}{2} \bigl\Vert x(t) - z \bigr\Vert ^{2}. \end{aligned} \end{aligned}$$

In the following theorem, we formulate sufficient conditions that guarantee the decay of the energy of the the dynamical system (1) and discuss some of its consequences.

### Theorem 2

*Suppose that* $\alpha \geq 1$, *λ* *is nondecreasing on* $[t_{0},+\infty )$ *and the following conditions*

11 $$ b(t) > \dot{\beta}(t) + \frac{\beta (t)}{t}\quad \textit{for every } t \geq t_{0} $$

*and*

12 $$ (\alpha - 3) w(t) - t \dot{w}(t) \geq 0 \quad \textit{for every } t \geq t_{0} $$

*are satisfied*. *Then*, *for a solution* $x : [t_{0},+\infty ) \rightarrow H$ *to* (1), *the following statements are true*:

(i) $\dot{E}_{c} (t) \leq 0$ *for every* $t \geq t_{0}$;
(ii) $\Phi _{\lambda (t)} (x(t)) - \Phi ^{*} \leq \frac{E_{\alpha - 1}(t_{0})}{t^{2}w(t)} $ *for every* $t \geq t_{0}$;
(iii) $\int _{t_{0}}^{+\infty} ( t^{2} w(t) \frac{\dot{\lambda}(t)}{2} + t^{2} \beta (t) w(t) ) \| \nabla \Phi _{\lambda (t)} (x(t)) \|^{2} \,dt < +\infty $;
(iv) $\int _{t_{0}}^{+\infty} ( (\alpha - 3)tw(t) - t^{2} \dot{w}(t) ) (\Phi _{\lambda (t)} (x(t)) - \Phi ^{*}) \,dt < +\infty $.
  *Moreover*, *assuming that* $\alpha > 1$ *and that*
  13 $$ \textit{there exists } \varepsilon \in (0, \alpha - 1) \textit{ such that } (\alpha - 3) w(t) - t \dot{w}(t) \geq \varepsilon b(t) \quad \forall t \geq t_{0}, $$
  *it holds*;
(v) $\int _{t_{0}}^{+\infty} t \| \dot{x}(t) \|^{2} \,dt < +\infty $;
(vi) *the trajectory* *x* *is bounded and*
(vii) $\int _{t_{0}}^{+\infty} t b(t) (\Phi _{\lambda (t)} (x(t)) - \Phi ^{*}) \,dt < +\infty $.

### Proof

For every $t \geq t_{0}$, we obtain

$$\begin{aligned} \dot{E}_{c}(t) & = \bigl( 2tw(t) + t^{2} \dot{w}(t) + \beta (t) ( \alpha -1-c) + (\alpha -1-c) t \dot{\beta}(t) \bigr) \bigl(\Phi _{\lambda (t)} \bigl(x(t)\bigr) - \Phi ^{*}\bigr) \\ &\quad{} + \bigl( t^{2} w(t) + \beta (t) t (\alpha -1-c) \bigr) \biggl( \bigl\langle \nabla \Phi _{\lambda (t)} \bigl(x(t)\bigr), \dot{x}(t) \bigr\rangle - \frac{\dot{\lambda}(t)}{2} \bigl\Vert \nabla \Phi _{\lambda (t)} \bigl(x(t)\bigr) \bigr\Vert ^{2} \biggr) \\ &\quad{} + \biggl\langle c\bigl(x(t) - z\bigr) + t \dot{x}(t) + t \beta (t) \nabla \Phi _{ \lambda (t)} \bigl(x(t)\bigr), (c + 1) \dot{x}(t) + t \ddot{x}(t) \\ &\quad{} + + t \beta (t) \frac{d}{dt} \bigl( \nabla \Phi _{\lambda (t)} \bigl(x(t)\bigr) \bigr)\bigl(\beta (t) + t \dot{\beta}(t)\bigr) \nabla \Phi _{\lambda (t)} \bigl(x(t)\bigr) \biggr\rangle \\ &\quad {}+ c(\alpha - 1 - c) \bigl\langle x(t) - z, \dot{x}(t) \bigr\rangle , \end{aligned}$$

where we used that

14 $$ \frac{d}{dt} \bigl( \Phi _{\lambda (t)}\bigl(x(t) \bigr) - \Phi ^{*} \bigr) = \bigl\langle \nabla \Phi _{\lambda (t)} \bigl(x(t)\bigr), \dot{x}(t) \bigr\rangle - \frac{\dot{\lambda}(t)}{2} \bigl\Vert \nabla \Phi _{\lambda (t)} x(t) \bigr\Vert ^{2}. $$

Using (1) to replace $\ddot{x}(t) $, we may write the third summand in the formulation of $\dot{E}_{c}(t)$ for every $t \geq t_{0}$ as

$$\begin{aligned} & \bigl\langle c\bigl(x(t) - z\bigr) + t \dot{x}(t) + t \beta (t) \nabla \Phi _{ \lambda (t)} \bigl(x(t)\bigr), \\ &\qquad {}(c + 1 - \alpha ) \dot{x}(t)+ \bigl(\beta (t) + t \dot{\beta}(t) - tb(t)\bigr) \nabla \Phi _{\lambda (t)} \bigl(x(t)\bigr) \bigr\rangle \\ & \quad = c(c + 1 - \alpha ) \bigl\langle x(t) - z, \dot{x}(t) \bigr\rangle + c \bigl( \beta (t) + t \dot{\beta}(t) - tb(t) \bigr) \bigl\langle x(t) - z, \nabla \Phi _{\lambda (t)} \bigl(x(t)\bigr) \bigr\rangle \\ &\qquad {}+ (c + 1 - \alpha )t \bigl\Vert \dot{x}(t) \bigr\Vert ^{2} + \bigl( \beta (t) + t \dot{\beta}(t) - tb(t) \bigr) t \bigl\langle \dot{x}(t), \nabla \Phi _{\lambda (t)}\bigl(x(t)\bigr) \bigr\rangle \\ &\quad \quad{} + t \beta (t) (c + 1 - \alpha ) \bigl\langle \dot{x}(t), \nabla \Phi _{\lambda (t)} \bigl(x(t)\bigr) \bigr\rangle + t \beta (t) \bigl(\beta (t) + t \dot{\beta}(t) - tb(t)\bigr) \bigl\Vert \nabla \Phi _{\lambda (t)} \bigl(x(t)\bigr) \bigr\Vert ^{2}. \end{aligned}$$

Overall, since $\beta (t) + t \dot{\beta}(t) - t b(t) = -t w(t)$, we obtain for every $t \geq t_{0}$

$$\begin{aligned} \dot{E}_{c}(t) & = \bigl( 2tw(t) + t^{2} \dot{w}(t) - \bigl(\beta (t) + t \dot{\beta}(t)\bigr) (c + 1 - \alpha ) \bigr) \bigl(\Phi _{\lambda (t)} \bigl(x(t)\bigr) - \Phi ^{*}\bigr) \\ &\quad{} + \bigl( t^{2} w(t) - t \beta (t) (c + 1 - \alpha ) \bigr) \biggl( \bigl\langle \nabla \Phi _{\lambda (t)} \bigl(x(t)\bigr), \dot{x}(t) \bigr\rangle - \frac{\dot{\lambda}(t)}{2} \bigl\Vert \nabla \Phi _{\lambda (t)} \bigl(x(t)\bigr) \bigr\Vert ^{2} \biggr) \\ &\quad{} - c t w(t) \bigl\langle x(t) - z, \nabla \Phi _{\lambda (t)} \bigl(x(t) \bigr) \bigr\rangle + (c + 1 - \alpha )t \bigl\Vert \dot{x}(t) \bigr\Vert ^{2} - t^{2} w(t) \bigl\langle \dot{x}(t), \nabla \Phi _{\lambda (t)}\bigl(x(t)\bigr) \bigr\rangle \\ &\quad{} + t \beta (t) (c + 1 - \alpha ) \bigl\langle \dot{x}(t), \nabla \Phi _{\lambda (t)} \bigl(x(t)\bigr) \bigr\rangle - t^{2} \beta (t) w(t) \bigl\Vert \nabla \Phi _{\lambda (t)} \bigl(x(t)\bigr) \bigr\Vert ^{2}. \end{aligned}$$

Notice that the terms with $\langle \nabla \Phi _{\lambda (t)} (x(t)), \dot{x}(t) \rangle $ cancel each other; thus, after simplification, we obtain for every $t \geq t_{0}$

15 $$ \begin{aligned} \dot{E}_{c}(t) & = \bigl( 2tw(t) + t^{2} \dot{w}(t) + \bigl( \beta (t) + t \dot{\beta}(t)\bigr) (\alpha -1 -c) \bigr) \bigl(\Phi _{\lambda (t)} \bigl(x(t)\bigr) - \Phi ^{*}\bigr) \\ &\quad{} - \bigl( t^{2} w(t) + t \beta (t) (\alpha -1 -c) \bigr) \frac{\dot{\lambda}(t)}{2} \bigl\Vert \nabla \Phi _{\lambda (t)} \bigl(x(t)\bigr) \bigr\Vert ^{2} \\ &\quad {}- c t w(t) \bigl\langle x(t) - z, \nabla \Phi _{\lambda (t)} \bigl(x(t)\bigr) \bigr\rangle \\ &\quad{} - (\alpha -1-c)t \bigl\Vert \dot{x}(t) \bigr\Vert ^{2} - t^{2} \beta (t) w(t) \bigl\Vert \nabla \Phi _{\lambda (t)} \bigl(x(t)\bigr) \bigr\Vert ^{2}. \end{aligned} $$

Thanks to (11), $w(t)$ is positive for every $t \geq t_{0}$, thus

$$ -c t w(t) \bigl\langle x(t) - z, \nabla \Phi _{\lambda (t)} \bigl(x(t)\bigr) \bigr\rangle \leq -c t w(t) \bigl(\Phi _{\lambda (t)}\bigl(x(t)\bigr) - \Phi ^{*}\bigr), $$

which leads to

16 $$ \begin{aligned} \dot{E}_{c}(t) & \leq \bigl( (2 - c)tw(t) + t^{2} \dot{w}(t) + \bigl(\beta (t) + t \dot{\beta}(t)\bigr) (\alpha -1 -c) \bigr) \bigl( \Phi _{\lambda (t)} \bigl(x(t)\bigr) - \Phi ^{*} \bigr) \\ &\quad{} - \biggl( \bigl( t^{2} w(t) + t \beta (t) (\alpha - 1 - c) \bigr) \frac{\dot{\lambda}(t)}{2} + t^{2} \beta (t) w(t) \biggr) \bigl\Vert \nabla \Phi _{\lambda (t)} \bigl(x(t)\bigr) \bigr\Vert ^{2} \\ &\quad {}- ( \alpha - c - 1)t \bigl\Vert \dot{x}(t) \bigr\Vert ^{2}. \end{aligned} $$

By (10) and the fact that *λ* is nondecreasing, we deduce that

$$ \bigl( t^{2} w(t) + t \beta (t) (\alpha -1 -c) \bigr) \frac{\dot{\lambda}(t)}{2} + t^{2} \beta (t) w(t) \geq 0, $$

so we obtain for every $t \geq t_{0}$

17 $$ \begin{aligned} \dot{E}_{c}(t) & \leq \bigl( (2 - c)tw(t) + t^{2} \dot{w}(t) + \bigl(\beta (t) + t \dot{\beta}(t)\bigr) (\alpha - 1 - c) \bigr) \bigl( \Phi _{\lambda (t)} \bigl(x(t)\bigr) - \Phi ^{*}\bigr) \\ &\quad{} - \biggl( \bigl( t^{2} w(t) + t \beta (t) (\alpha - c - 1) \bigr) \frac{\dot{\lambda}(t)}{2} + t^{2} \beta (t) w(t) \biggr) \bigl\Vert \nabla \Phi _{\lambda (t)} \bigl(x(t)\bigr) \bigr\Vert ^{2}. \end{aligned} $$

Let us choose $c := \alpha - 1$. According to (12), we obtain for the coefficient of $\Phi _{\lambda (t)} (x(t)) - \Phi ^{*}$ in (17)

$$ (2 - c)tw(t) + t^{2} \dot{w}(t) + \bigl(\beta (t) + t \dot{\beta}(t) \bigr) ( \alpha - 1 - c) = -t \bigl( (\alpha -3) w(t) - t \dot{w}(t) \bigr) \leq 0. $$

Therefore, (17) allows us to deduce for every $t \geq t_{0}$

$$\begin{aligned} \dot{E}_{\alpha - 1}(t) & \leq - \bigl( (\alpha -3)tw(t) - t^{2} \dot{w}(t) \bigr) \bigl(\Phi _{\lambda (t)} \bigl(x(t)\bigr) - \Phi ^{*}\bigr) \\ &\quad {}- \biggl( t^{2} w(t) \frac{\dot{\lambda}(t)}{2} + t^{2} \beta (t) w(t) \biggr) \bigl\Vert \nabla \Phi _{\lambda (t)} \bigl(x(t)\bigr) \bigr\Vert ^{2} \\ & \leq 0. \end{aligned}$$

We have just established that $E_{\alpha -1}$ is nonincreasing, which for every $t\geq t_{0}$ leads to

$$\begin{aligned} E_{\alpha - 1}(t) &= t^{2} w(t) \bigl(\Phi _{\lambda (t)} \bigl(x(t)\bigr) - \Phi ^{*}\bigr) + \frac{1}{2} \bigl\Vert (\alpha - 1) \bigl(x(t) - z\bigr) + t \dot{x}(t) + t \beta (t) \nabla \Phi _{\lambda (t)} \bigl(x(t)\bigr) \bigr\Vert ^{2} \\ &\leq E_{\alpha - 1} (t_{0}). \end{aligned}$$

From here, we obtain for every $t\geq t_{0}$

18 $$ \Phi _{\lambda (t)} \bigl(x(t)\bigr) - \Phi ^{*} \leq \frac{E_{\alpha - 1}(t_{0})}{t^{2}w(t)}, $$

which proves (ii). Moreover, by integration, we obtain

19 $$ \int _{t_{0}}^{+\infty} \biggl( t^{2} w(t) \frac{\dot{\lambda}(t)}{2} + t^{2} \beta (t) w(t) \biggr) \bigl\Vert \nabla \Phi _{\lambda (t)} \bigl(x(t)\bigr) \bigr\Vert ^{2} \,dt \leq E_{\alpha -1}(t_{0}) < +\infty $$

and

20 $$ \int _{t_{0}}^{+\infty} \bigl( (\alpha - 3)tw(t) - t^{2} \dot{w}(t) \bigr) \bigl(\Phi _{\lambda (t)} \bigl(x(t)\bigr) - \Phi ^{*}\bigr) \,dt \leq E_{\alpha - 1}(t_{0}) < + \infty , $$

which are the claims (iii) and (iv).

From now on, we assume that $\alpha > 1$ and choose $c := \alpha - 1 - \varepsilon $, where *ε* is given by (13). In this setting, (16) reads for every $t \geq t_{0}$,

21 $$\begin{aligned} \begin{aligned} \dot{E}_{\alpha - 1 - \varepsilon}(t) & \leq \bigl( (3 - \alpha + \varepsilon )tw(t) + t^{2} \dot{w}(t) + \varepsilon \bigl(\beta (t) + t \dot{\beta}(t)\bigr) \bigr) \bigl(\Phi _{\lambda (t)} \bigl(x(t)\bigr) - \Phi ^{*}\bigr) \\ &\quad{} - \biggl( \bigl( t^{2} w(t) + \varepsilon t \beta (t) \bigr) \frac{\dot{\lambda}(t)}{2} + t^{2} \beta (t) w(t) \biggr) \bigl\Vert \nabla \Phi _{\lambda (t)} \bigl(x(t)\bigr) \bigr\Vert ^{2} - \varepsilon t \bigl\Vert \dot{x}(t) \bigr\Vert ^{2} \\ & = - t \bigl( (\alpha -3)w(t) - t \dot{w}(t) - \varepsilon b(t) \bigr) \bigl( \Phi _{\lambda (t)} \bigl(x(t)\bigr) - \Phi ^{*}\bigr) \\ &\quad{} - \biggl( \bigl( t^{2} w(t) + \varepsilon t \beta (t) \bigr) \frac{\dot{\lambda}(t)}{2} + t^{2} \beta (t) w(t) \biggr) \bigl\Vert \nabla \Phi _{\lambda (t)} \bigl(x(t)\bigr) \bigr\Vert ^{2} - \varepsilon t \bigl\Vert \dot{x}(t) \bigr\Vert ^{2}. \end{aligned} \end{aligned}$$

So, under the condition (13), $\dot{E}_{\alpha - 1 - \varepsilon}(t) \leq 0$ for every $t \geq t_{0}$. Integrating (21), we obtain

22 $$ \int _{t_{0}}^{+\infty} t \bigl\Vert \dot{x}(t) \bigr\Vert ^{2} \,dt < +\infty , $$

which gives the claim (v). From the fact that the energy function

$$\begin{aligned} E_{\alpha - 1 - \varepsilon}(t) & = \bigl( t^{2} w(t) + \varepsilon t \beta (t) \bigr) \bigl(\Phi _{\lambda (t)} \bigl(x(t)\bigr) - \Phi ^{*} \bigr) \\ &\quad{} + \frac{1}{2} \bigl\Vert (\alpha - 1 - \varepsilon ) \bigl(x(t) - z \bigr) + t \dot{x}(t) + t \beta (t) \nabla \Phi _{\lambda (t)} \bigl(x(t)\bigr) \bigr\Vert ^{2} \\ &\quad {}+ \frac{(\alpha - 1 - \varepsilon )\varepsilon}{2} \bigl\Vert x(t) - z \bigr\Vert ^{2} \end{aligned}$$

is bounded from above, and it is nonnegative on $[t_{0}, +\infty )$, it follows that the trajectory *x* is bounded, which is item (vi). Finally, from (13) and (20), we deduce the claim (vii)

23 $$ \begin{aligned} &\int _{t_{0}}^{+\infty} \varepsilon t b(t) \bigl(\Phi _{\lambda (t)} \bigl(x(t)\bigr) - \Phi ^{*}\bigr) \,dt \\ &\quad \leq \int _{t_{0}}^{+\infty} \bigl( (\alpha - 3)tw(t) - t^{2} \dot{w}(t) \bigr) \bigl(\Phi _{\lambda (t)} \bigl(x(t)\bigr) - \Phi ^{*}\bigr) \,dt < +\infty , \end{aligned} $$

which finishes the proof. □

The following auxiliary result will be needed later.

### Lemma 3

*Suppose that* $\alpha >1$ *and* (13) *holds*, *that* *λ* *and* *β* *are nondecreasing on* $[t_{0}, +\infty )$, *and that* (11) *holds*. *Then*, *for a solution* $x : [t_{0},+\infty ) \rightarrow H$ *to* (1), *it holds*

24 $$ \int _{t_{0}}^{+\infty} t w(t) \bigl\langle \nabla \Phi _{\lambda (t)} \bigl(x(t)\bigr), x(t) - z \bigr\rangle \,dt < +\infty . $$

### Proof

Recall that according to (15), we have for every $t \geq t_{0}$

$$\begin{aligned} \dot{E}_{c}(t) & = \bigl( 2tw(t) + t^{2} \dot{w}(t) + \bigl(\beta (t) + t \dot{\beta}(t)\bigr) (\alpha - 1 - c) \bigr) \bigl(\Phi _{\lambda (t)} \bigl(x(t)\bigr) - \Phi ^{*}\bigr) \\ &\quad{} - \bigl( t^{2} w(t) + t \beta (t) (\alpha -1-c) \bigr) \frac{\dot{\lambda}(t)}{2} \bigl\Vert \nabla \Phi _{\lambda (t)} \bigl(x(t)\bigr) \bigr\Vert ^{2} \\ &\quad{} - c t w(t) \bigl\langle x(t) - z, \nabla \Phi _{\lambda (t)} \bigl(x(t) \bigr) \bigr\rangle - (\alpha - 1 - c)t \bigl\Vert \dot{x}(t) \bigr\Vert ^{2} \\ &\quad {}- t^{2} \beta (t) w(t) \bigl\Vert \nabla \Phi _{\lambda (t)} \bigl(x(t)\bigr) \bigr\Vert ^{2}. \end{aligned}$$

We choose again $c: = \alpha - 1$ and split the term $(\alpha - 1)t w(t) \langle x(t) - z, \nabla \Phi _{\lambda (t)} (x(t)) \rangle $ into the sum of two expressed in terms of *ε* given by (13). For every $t \geq t_{0}$, we have

$$\begin{aligned} \dot{E}_{\alpha - 1}(t) & \leq \bigl( 2tw(t) + t^{2} \dot{w}(t) \bigr) \bigl( \Phi _{\lambda (t)} \bigl(x(t)\bigr) - \Phi ^{*} \bigr) \\ &\quad{} - ( \alpha - 1 - \varepsilon ) t w(t) \bigl\langle x(t) - z, \nabla \Phi _{\lambda (t)} \bigl(x(t)\bigr) \bigr\rangle - \varepsilon t w(t) \bigl\langle x(t) - z, \nabla \Phi _{\lambda (t)} \bigl(x(t)\bigr) \bigr\rangle . \end{aligned}$$

By applying the convex subdifferential inequality, we obtain for every $t \geq t_{0}$

25 $$\begin{aligned} \begin{aligned} \dot{E}_{\alpha - 1}(t) & \leq \bigl( 2tw(t) + t^{2} \dot{w}(t) - ( \alpha - 1 - \varepsilon ) t w(t) \bigr) \bigl( \Phi _{\lambda (t)} \bigl(x(t)\bigr) - \Phi ^{*}\bigr) \\ &\quad {} - \varepsilon t w(t) \bigl\langle x(t) - z, \nabla \Phi _{ \lambda (t)} \bigl(x(t) \bigr) \bigr\rangle \\ & = \bigl( t^{2} \dot{w}(t) - ( \alpha - 3 - \varepsilon ) t w(t) \bigr) \bigl(\Phi _{\lambda (t)} \bigl(x(t)\bigr) - \Phi ^{*}\bigr) \\ &\quad {}- \varepsilon t w(t) \bigl\langle x(t) - z, \nabla \Phi _{\lambda (t)} \bigl(x(t)\bigr) \bigr\rangle . \end{aligned} \end{aligned}$$

Since *β* is nondecreasing, for every $t \geq t_{0}$, it holds

$$ b(t) = w(t) + \dot{\beta}(t) + \frac{\beta (t)}{t} \geq w(t), $$

thus, (13) leads to $t^{2} \dot{w}(t) - (\alpha - 3 -\varepsilon ) t w(t) \leq 0$. Consequently, we obtain from (25) by integration

$$ \int _{t_{0}}^{+\infty} t w(t) \bigl\langle \nabla \Phi _{\lambda (t)} \bigl(x(t)\bigr), x(t) - z \bigr\rangle \,dt \leq \frac{E_{\alpha - 1}(t_{0})}{\varepsilon} < +\infty . $$

 □

Now, we are in a position to improve the convergence rates we obtained previously in (18) and derive from here convergence rates for Φ.

### Theorem 4

*Suppose that* $\alpha >1$ *and* (13) *holds*, *that* *λ* *and* *β* *are nondecreasing on* $[t_{0}, +\infty )$, *and that* (11) *holds*. *In addition*, *assume that*

26 $$ \int _{t_{0}}^{+\infty} \biggl[ \frac{ (\dot{\lambda}(t) )^{2} t^{3} \beta ^{2}(t)}{\lambda ^{4}(t)} - \frac{\dot{\lambda}(t) t^{2} b(t)}{2 \lambda ^{2}(t)} \biggr]_{+} \,dt < +\infty , $$

*where* $[\cdot ]_{+}$ *denotes the positive part of the expression inside the brackets*, *and that there exists* $C>0$ *such that*

27 $$ \frac{d}{dt} \bigl( t^{2} b(t) \bigr) \leq C t b(t) \quad \textit{for every } t \geq t_{0}. $$

*Then*, *for a solution* $x : [t_{0},+\infty ) \rightarrow H$ *to* (1), *it holds*

28 $$ \Phi _{\lambda (t)}\bigl(x(t)\bigr) - \Phi ^{*} = o \biggl( \frac{1}{t^{2} b(t)} \biggr) \quad \textit{and}\quad \bigl\Vert \dot{x}(t) \bigr\Vert = o \biggl( \frac{1}{t} \biggr)\quad \textit{as } t \to +\infty . $$

*Moreover*,

29 $$ \bigl\Vert \nabla \Phi _{\lambda (t)} \bigl(x(t)\bigr) \bigr\Vert = o \biggl( \frac{1}{t \sqrt{b(t) \lambda (t)}} \biggr) \quad \textit{as } t \to + \infty , $$

*and*

30 $$ \begin{aligned} &\Phi \bigl(\operatorname{prox}_{\lambda (t) \Phi} \bigl(x(t)\bigr)\bigr) - \Phi ^{*} = o \biggl( \frac{1}{t^{2} b(t)} \biggr) \quad \textit{and} \\ &\bigl\Vert \operatorname{prox}_{ \lambda (t) \Phi} \bigl(x(t)\bigr) - x(t) \bigr\Vert = o \biggl( \frac{\sqrt{\lambda (t)}}{t \sqrt{b(t)}} \biggr) \quad \textit{as } t \to + \infty . \end{aligned} $$

### Proof

First, we notice that for every $t \geq t_{0}$, it holds

$$\begin{aligned} \biggl\langle \frac{d}{dt} \bigl( \nabla \Phi _{\lambda (t)} \bigl(x(t)\bigr) \bigr), \dot{x}(t) \biggr\rangle &= \biggl\langle \lim _{h \to 0} \frac{\nabla \Phi _{\lambda (t + h)} (x(t + h)) - \nabla \Phi _{\lambda (t)} (x(t))}{h}, \dot{x}(t) \biggr\rangle \\ &= \biggl\langle \lim_{h \to 0} \frac{\nabla \Phi _{\lambda (t + h)} (x(t + h)) - \nabla \Phi _{\lambda (t + h)} (x(t))}{h}, \dot{x}(t) \biggr\rangle \\ &\quad{}+ \biggl\langle \lim_{h \to 0} \frac{\nabla \Phi _{\lambda (t + h)} (x(t)) - \nabla \Phi _{\lambda (t)} (x(t))}{h}, \dot{x}(t) \biggr\rangle . \end{aligned}$$

For every $h > 0$, by the monotonicity of the gradient of a convex function, we have

$$ \biggl\langle \frac{\nabla \Phi _{\lambda (t + h)} (x(t + h)) - \nabla \Phi _{\lambda (t + h)} (x(t))}{h}, \frac{x(t + h) - x(t)}{h} \biggr\rangle \geq 0, $$

so letting *h* tend to zero, we obtain

$$ \biggl\langle \lim_{h \to 0} \frac{\nabla \Phi _{\lambda (t + h)} (x(t + h)) - \nabla \Phi _{\lambda (t + h)} (x(t))}{h}, \dot{x}(t) \biggr\rangle \geq 0. $$

Consequently, for every $t \geq t_{0}$, it holds

$$\begin{aligned} & \biggl\langle \frac{d}{dt} \bigl( \nabla \Phi _{\lambda (t)} \bigl(x(t)\bigr) \bigr), \dot{x}(t) \biggr\rangle \\ &\quad \geq \biggl\langle \lim_{h \to 0} \frac{\nabla \Phi _{\lambda (t + h)} (x(t)) - \nabla \Phi _{\lambda (t)} (x(t))}{h}, \dot{x}(t) \biggr\rangle \\ &\quad = \lim_{h \to 0} \biggl\langle \frac{ (\lambda (t + h)) \operatorname{prox}_{\lambda (t) \Phi} (x(t)) - \lambda (t) \operatorname{prox}_{(\lambda (t + h)) \Phi} (x(t)) - ( \lambda (t + h) - \lambda (t) ) x(t) }{\lambda (t) \lambda (t + h)h}, \dot{x}(t) \biggr\rangle \\ &\quad = \lim_{h \to 0} \biggl\langle \frac{ ( \lambda (t + h) - \lambda (t) ) ( \operatorname{prox}_{\lambda (t) \Phi} (x(t)) - x(t) )}{\lambda (t) \lambda (t + h) h}, \dot{x}(t) \biggr\rangle \\ &\quad \quad{} - \lim_{h \to 0} \biggl\langle \frac{\operatorname{prox}_{(\lambda (t + h)) \Phi} (x(t)) - \operatorname{prox}_{\lambda (t) \Phi} (x(t))}{\lambda (t+h)h}, \dot{x}(t) \biggr\rangle \\ &\quad \geq \frac{\dot{\lambda}(t)}{\lambda ^{2}(t)} \bigl\langle \operatorname{prox}_{\lambda (t) \Phi} \bigl(x(t) \bigr) - x(t), \dot{x}(t) \bigr\rangle - \lim_{h \to 0} \frac{(\lambda (t + h) - \lambda (t)) \Vert \nabla \Phi _{\lambda (t)} (x(t)) \Vert \Vert \dot{x}(t) \Vert }{\lambda (t + h) h} \\ &\quad = \frac{\dot{\lambda}(t)}{\lambda ^{2}(t)} \bigl\langle \operatorname{prox}_{\lambda (t) \Phi} \bigl(x(t)\bigr) - x(t), \dot{x}(t) \bigr\rangle - \frac{\dot{\lambda}(t) \Vert \nabla \Phi _{\lambda (t)} (x(t)) \Vert \Vert \dot{x}(t) \Vert }{\lambda (t)} \\ &\quad = - \frac{\dot{\lambda}(t)}{\lambda (t)} \bigl\langle \nabla \Phi _{\lambda (t)} \bigl(x(t) \bigr), \dot{x}(t) \bigr\rangle - \frac{\dot{\lambda}(t) \Vert \nabla \Phi _{\lambda (t)} (x(t)) \Vert \Vert \dot{x}(t) \Vert }{\lambda (t)} \\ &\quad \geq - \frac{2 \dot{\lambda}(t) \Vert \nabla \Phi _{\lambda (t)} (x(t)) \Vert \Vert \dot{x}(t) \Vert }{\lambda (t)}, \end{aligned}$$

where we used (6), (7), and the Cauchy–Schwarz inequality. Now, we multiply (1) by $t^{2} \dot{x}(t)$ to deduce, using the inequality above and (14), for every $t \geq t_{0}$

$$\begin{aligned} 0 & = t^{2} \bigl\langle \ddot{x}(t), \dot{x}(t) \bigr\rangle + \alpha t \bigl\Vert \dot{x}(t) \bigr\Vert ^{2} + t^{2} \beta (t) \biggl\langle \frac{d}{dt} \bigl( \nabla \Phi _{\lambda (t)} \bigl(x(t)\bigr) \bigr), \dot{x}(t) \biggr\rangle \\ &\quad {}+ t^{2} b(t) \bigl\langle \nabla \Phi _{\lambda (t)}\bigl(x(t)\bigr), \dot{x}(t) \bigr\rangle \\ & \geq t^{2} \frac{d}{dt} \biggl( \frac{1}{2} \bigl\Vert \dot{x}(t) \bigr\Vert ^{2} \biggr) + \alpha t \bigl\Vert \dot{x}(t) \bigr\Vert ^{2} + t^{2} b(t) \frac{d}{dt} \bigl( \Phi _{\lambda (t)}\bigl(x(t)\bigr) - \Phi ^{*} \bigr) \\ &\quad{}+ \frac{\dot{\lambda}(t) t^{2} b(t)}{2} \bigl\Vert \nabla \Phi _{\lambda (t)} x(t) \bigr\Vert ^{2} - \frac{2 t^{2} \beta (t) \dot{\lambda}(t)}{\lambda (t)} \bigl\Vert \nabla \Phi _{\lambda (t)} \bigl(x(t) \bigr) \bigr\Vert \bigl\Vert \dot{x}(t) \bigr\Vert \\ & \geq \frac{d}{dt} \biggl( \frac{t^{2}}{2} \bigl\Vert \dot{x}(t) \bigr\Vert ^{2} + t^{2} b(t) \bigl(\Phi _{\lambda (t)}\bigl(x(t)\bigr) - \Phi ^{*}\bigr) \biggr) + (\alpha - 1) t \bigl\Vert \dot{x}(t) \bigr\Vert ^{2} \\ &\quad {}- \bigl( \Phi _{\lambda (t)}\bigl(x(t)\bigr) - \Phi ^{*} \bigr) \frac{d}{dt} \bigl( t^{2} b(t) \bigr) \\ &\quad{} - \biggl\{ \biggl[ \biggl( \frac{\dot{\lambda}(t)}{\lambda (t)} \biggr)^{2} t^{3} \beta ^{2}(t) - \frac{\dot{\lambda}(t) t^{2} b(t)}{2} \biggr] \bigl\Vert \nabla \Phi _{ \lambda (t)} \bigl(x(t)\bigr) \bigr\Vert ^{2} + t \bigl\Vert \dot{x}(t) \bigr\Vert ^{2} \biggr\} . \end{aligned}$$

Using (27), we obtain for every $t \geq t_{0}$

$$\begin{aligned} &\frac{d}{dt} \biggl( \frac{t^{2}}{2} \bigl\Vert \dot{x}(t) \bigr\Vert ^{2} + t^{2} b(t) \bigl( \Phi _{\lambda (t)} \bigl(x(t)\bigr) - \Phi ^{*}\bigr) \biggr) \\ &\quad \leq [2 - \alpha ]_{+} t \bigl\Vert \dot{x}(t) \bigr\Vert ^{2} + \bigl( \Phi _{\lambda (t)}\bigl(x(t)\bigr) - \Phi ^{*} \bigr) C t b(t) \\ &\quad \quad{} + \biggl[ \biggl( \frac{\dot{\lambda}(t)}{\lambda (t)} \biggr)^{2} t^{3} \beta ^{2}(t) - \frac{\dot{\lambda}(t) t^{2} b(t)}{2} \biggr]_{+} \bigl\Vert \nabla \Phi _{\lambda (t)} \bigl(x(t) \bigr) \bigr\Vert ^{2}. \end{aligned}$$

Next, we show the integrability of the right-hand side of the expression above. The first term is integrable according to Theorem 2 (v), and the second one is integrable according to Theorem 2 (vii). Further, since

$$ \bigl\Vert \nabla \Phi _{\lambda (t)}\bigl(x(t)\bigr) - \nabla \Phi _{\lambda (t)}(z) \bigr\Vert \leq \frac{1}{\lambda (t)} \bigl\Vert x(t) - z \bigr\Vert \quad \forall t \geq t_{0}, $$

and taking into the account the boundedness of the trajectory *x* established in Theorem 2 (vi) and that $z \in \operatorname*{argmin}\Phi $, we deduce

$$ \bigl\Vert \nabla \Phi _{\lambda (t)}\bigl(x(t)\bigr) \bigr\Vert = O \biggl( \frac{1}{\lambda (t)} \biggr)\quad \text{as } t \to +\infty . $$

So, under the assumption (26), we obtain that there exists $\widetilde{C} > 0$ such that for every $t \geq t_{0}$

$$\begin{aligned} \begin{aligned} &\int _{t_{0}}^{t} \biggl[ \biggl( \frac{\dot{\lambda}(s)}{\lambda (s)} \biggr)^{2} s^{3} \beta ^{2}(s) - \frac{\dot{\lambda}(s) s^{2} b(s)}{2} \biggr]_{+} \bigl\Vert \nabla \Phi _{ \lambda (s)} \bigl(x(s)\bigr) \bigr\Vert ^{2} \,ds \\ &\quad \leq \widetilde{C} \int _{t_{0}}^{t} \biggl[ \frac{ (\dot{\lambda}(s) )^{2} s^{3} \beta ^{2}(s)}{\lambda ^{4}(s)} - \frac{\dot{\lambda}(s) s^{2} b(s)}{2 \lambda ^{2}(s)} \biggr]_{+} \,ds \\ &\quad < +\infty . \end{aligned} \end{aligned}$$

Applying Lemma 6 in the Appendix, we conclude that the following limit

$$ L := \lim_{t \to +\infty} \biggl( \frac{t^{2}}{2} \bigl\Vert \dot{x}(t) \bigr\Vert ^{2} + t^{2} b(t) \bigl(\Phi _{\lambda (t)}\bigl(x(t)\bigr) - \Phi ^{*}\bigr) \biggr) \geq 0 $$

exists. We will show that $L = 0$. Supposing that $L >0$, we deduce that there exists $t^{*} \geq t_{0}$ such that for every $t \geq t^{*}$

$$ \frac{t}{2} \bigl\Vert \dot{x}(t) \bigr\Vert ^{2} + t b(t) \bigl(\Phi _{\lambda (t)}\bigl(x(t)\bigr) - \Phi ^{*}\bigr) \geq \frac{L}{2t}. $$

Integrating the last inequality on $[t^{*}, +\infty )$, we arrive at the contradiction with the integrability of the left-hand side as proved in Theorem 2 (v) and (vii). Therefore, $L = 0$, and we obtain

$$ \Phi _{\lambda (t)}\bigl(x(t)\bigr) - \Phi ^{*} = o \biggl( \frac{1}{t^{2} b(t)} \biggr) \quad \text{and} \quad \bigl\Vert \dot{x}(t) \bigr\Vert = o \biggl( \frac{1}{t} \biggr) \quad \text{as } t \to +\infty . $$

Using the definition of the proximal mapping, we derive

31 $$ \begin{aligned} &\Phi _{\lambda (t)}\bigl(x(t)\bigr) - \Phi ^{*} \\ &\quad = \Phi \bigl(\operatorname{prox}_{ \lambda (t) \Phi}\bigl(x(t)\bigr)\bigr) - \Phi ^{*} + \frac{1}{2\lambda (t)} \bigl\Vert \operatorname{prox}_{\lambda (t) \Phi} \bigl(x(t)\bigr) - x(t) \bigr\Vert ^{2} \quad \forall t \geq t_{0}, \end{aligned} $$

which yields

$$ \begin{aligned} &\Phi \bigl(\operatorname{prox}_{\lambda (t) \Phi}\bigl(x(t)\bigr)\bigr) - \Phi ^{*} = o \biggl( \frac{1}{t^{2} b(t)} \biggr) \quad \text{and} \\ &\bigl\Vert \operatorname{prox}_{ \lambda (t) \Phi}\bigl(x(t)\bigr) - x(t) \bigr\Vert = o \biggl( \frac{\sqrt{\lambda (t)}}{t \sqrt{b(t)}} \biggr)\quad \text{as } t \to + \infty . \end{aligned} $$

According to (6), we obtain from here

$$ \bigl\Vert \nabla \Phi _{\lambda (t)} \bigl(x(t)\bigr) \bigr\Vert = o \biggl( \frac{1}{t \sqrt{b(t) \lambda (t)}} \biggr)\quad \text{as } t \to + \infty . $$

 □

## Convergence of the trajectories

In this section, we will investigate the weak convergence of the trajectory *x* to a minimizer of Φ.

### Theorem 5

*Suppose that* $\alpha >1$, (11) *and* (13) *hold*, *and* *λ* *and* *β* *are nondecreasing on* $[t_{0}, +\infty )$. *In addition*, *assume that*

32 $$ \lim_{t \to +\infty} \frac{\beta (t)}{t w(t)} = 0 $$

*and*

33 $$ \sup_{t \geq t_{0}} \frac{\lambda (t)}{t} < +\infty . $$

*If* $x : [t_{0},+\infty ) \rightarrow H$ *is a solution to* (1), *then* $x(t)$ *converges weakly to a minimizer of* Φ *as* $t \to +\infty $.

### Proof

Let $z \in \operatorname*{argmin}\Phi $. Previously, in Theorem 2, we established the existence of the limit of $E_{c}(t)$ as $t\to + \infty $ for $c = \alpha - 1$ and $c = \alpha - 1 - \varepsilon $, where $\varepsilon \in (0, \alpha -1)$ is given by (13). Thus, computing the difference

$$\begin{aligned} E_{\alpha - 1 - \varepsilon}(t) - E_{\alpha - 1}(t) & = \varepsilon t \beta (t) \bigl( \Phi _{\lambda (t)}\bigl(x(t)\bigr) - \Phi ^{*}\bigr) + \frac{\varepsilon (\alpha - 1)}{2} \bigl\Vert x(t) - z \bigr\Vert ^{2} \\ &\quad{} - \varepsilon \bigl\langle (\alpha - 1) \bigl(x(t) - z\bigr) + t (\dot{x}(t) + \beta (t) \nabla \Phi _{\lambda (t)}\bigl(x(t)\bigr), x(t) - z \bigr\rangle \\ & = \varepsilon t \beta (t) \bigl(\Phi _{\lambda (t)}\bigl(x(t)\bigr) - \Phi ^{*}\bigr) - \frac{\varepsilon (\alpha -1)}{2} \bigl\Vert x(t) - z \bigr\Vert ^{2} \\ &\quad{} - \varepsilon t \bigl\langle \dot{x}(t) + \beta (t) \nabla \Phi _{ \lambda (t)}\bigl(x(t)\bigr), x(t) - z \bigr\rangle , \end{aligned}$$

we deduce that the limit of the right-hand side exists. Thanks to (18), we derive for every $t \geq t_{0}$

$$ t \beta (t) \bigl(\Phi _{\lambda (t)}\bigl(x(t)\bigr) - \Phi ^{*} \bigr) \leq t \beta (t) \frac{E_{\alpha - 1}(t_{0})}{t^{2} w(t)} = E_{\alpha - 1}(t_{0}) \frac{\beta (t)}{t w(t)} $$

and from here, based on the assumption (32), we obtain

34 $$\begin{aligned} \lim_{t \to +\infty} t \beta (t) \bigl(\Phi _{\lambda (t)}\bigl(x(t)\bigr) - \Phi ^{*}\bigr) = 0. \end{aligned}$$

Hereby, we derived that the limit of the quantity

$$ p(t) := \frac{\alpha - 1}{2} \bigl\Vert x(t) - z \bigr\Vert ^{2} + t \bigl\langle \dot{x}(t), x(t) - z \bigr\rangle + t \beta (t) \bigl\langle \nabla \Phi _{\lambda (t)}\bigl(x(t)\bigr), x(t) - z \bigr\rangle $$

exists as $t \to +\infty $. Now, we are ready to prove the existence of the limit of $\| x(t) - z \|$ as $t \to +\infty $. Denote

$$ q(t) := \frac{\alpha - 1}{2} \bigl\Vert x(t) - z \bigr\Vert ^{2} + (\alpha - 1) \int _{t_{0}}^{t} \beta (s) \bigl\langle \nabla \Phi _{\lambda (s)}\bigl(x(s)\bigr), x(s) - z \bigr\rangle \,ds\quad \forall t \geq t_{0}. $$

For every $t \geq t_{0}$, it holds that

$$ p(t )= q(t) + \frac{t}{\alpha - 1} \dot{q}(t) - (\alpha - 1) \int _{t_{0}}^{t} \beta (s) \bigl\langle \nabla \Phi _{\lambda (s)}\bigl(x(s)\bigr), x(s) - z \bigr\rangle \,ds , $$

since

$$ \dot{q}(t) = (\alpha - 1) \bigl\langle x(t) - z, \dot{x}(t) \bigr\rangle + ( \alpha - 1 ) \bigl( \beta (s) \bigl\langle \nabla \Phi _{\lambda (s)}\bigl(x(s) \bigr), x(s) - z \bigr\rangle \bigr) $$

and

$$\begin{aligned} q(t) + \frac{t}{\alpha - 1} \dot{q}(t) & = \frac{\alpha - 1}{2} \bigl\Vert x(t) - z \bigr\Vert ^{2} + (\alpha - 1) \int _{t_{0}}^{t} \beta (s) \bigl\langle \nabla \Phi _{\lambda (s)}\bigl(x(s)\bigr), x(s) - z \bigr\rangle \,ds \\ &\quad{} + t \bigl\langle x(t) - z, \dot{x}(t) \bigr\rangle + t \bigl( \beta (s) \bigl\langle \nabla \Phi _{\lambda (s)}\bigl(x(s)\bigr), x(s) - z \bigr\rangle \bigr). \end{aligned}$$

By Lemma 3, we established that $\int _{t_{0}}^{+\infty} s w(s) \langle \nabla \Phi _{\lambda (s)}(x(s)), x(s) - z \rangle \,ds < +\infty $. In turn, (32) yields that

35 $$ \lim_{t \to +\infty} \int _{t_{0}}^{t} \beta (s) \bigl\langle \nabla \Phi _{\lambda (s)}\bigl(x(s)\bigr), x(s) - z \bigr\rangle \,ds \quad \text{exists}. $$

Finally,

$$ \lim_{t \to +\infty} \biggl( q(t) + \frac{t}{\alpha - 1} \dot{q}(t) \biggr)\quad \text{also exists}. $$

Applying now Lemma 7 in the Appendix, we immediately get the existence of the limit of $q(t)$ as $t \to +\infty $. By the definition of *q* and (35), we establish the first statement of Opial’s Lemma (see Lemma 8 in the Appendix), namely, that, for any $z \in \operatorname*{argmin}\Phi $,

$$ \lim_{t \to +\infty} \bigl\Vert x(t) - z \bigr\Vert \quad \text{exists}. $$

To establish the second term of Opial’s Lemma, first note that from (31) and (34), we have by denoting $\xi (t):=\operatorname{prox}_{\lambda (t)\Phi}(x(t))$, $\lim_{t \rightarrow +\infty} t\beta (t)(\Phi (\xi (t)) - \Phi ^{*}) = 0$ and $\lim_{t \rightarrow +\infty} \frac{t\beta (t)}{\lambda (t)} \| \xi (t) - x(t) \|^{2} = 0$. Using that *β* is nondecreasing and assumption (33), we deduce

$$ \lim_{t \to +\infty} \Phi \bigl(\xi (t)\bigr) = \Phi ^{*} \quad \text{and} \quad \lim_{t \to +\infty} \bigl\Vert \xi (t) - x(t) \bigr\Vert =0.$$

Considering a sequence $\{ t_{k} \}_{k \in \mathbb{N}} $ such that $\{x(t_{k})\}_{k \in \mathbb{N}} $ converges weakly to an element $z \in H $ as $k \to +\infty $, we notice that $\{\xi (t_{k})\}_{k \in \mathbb{N}} $ converges weakly to *z* as $k \to +\infty $. Now, the function Φ being convex and lower semicontinuous in the weak topology allows us to write

$$ \Phi (z) \leq \liminf_{k \to +\infty} \Phi \bigl(\xi (t_{k})\bigr) = \lim_{t \to +\infty} \Phi \bigl(\xi (t) \bigr) = \Phi ^{*}. $$

Hence, $z \in \operatorname*{argmin}\Phi $, and the second statement of Opial’s Lemma is shown. This gives the weak convergence of the trajectory $x(t)$ to a minimizer of Φ as $t \rightarrow +\infty $. □

### Remark 1

In the hypotheses of Theorem 4, to obtain the convergence of the trajectories, besides (33), it is enough to assume that

$$ \sup_{t \geq t_{0}} \frac{\beta (t)}{t w(t)} < +\infty $$

to guarantee (35). Indeed, in this case, (34) follows from the conclusion of Theorem 4

$$ \lim_{t \rightarrow +\infty} t \beta (t) \bigl(\Phi _{\lambda (t)} \bigl(x(t)\bigr) - \Phi ^{*}\bigr) \leq \lim_{t \rightarrow +\infty} t^{2} b(t) \bigl(\Phi _{ \lambda (t)}\bigl(x(t)\bigr) - \Phi ^{*}\bigr) \frac{\beta (t)}{t w(t)} =0.$$

### Remark 2

(Implicit discretization)

Implicit discretization of the dynamical system (1) with fixed step size $h >0$ leads to the numerical scheme that reads for every $k \geq 1$ (see also [7, 8])

$$ \frac{x_{k+1} - 2x_{k} + x_{k-1}}{h^{2}} + \frac{\alpha (x_{k+1} - x_{k})}{kh^{2}} + \frac{\beta _{k} ( \nabla \Phi _{\lambda _{k+1}} (x_{k+1}) - \nabla \Phi _{\lambda _{k}} (x_{k}) )}{h} + b_{k} \nabla \Phi _{\lambda _{k+1}} (x_{k+1}) = 0, $$

where $x_{k}$, $\lambda _{k}$, $\beta _{k}$, and $b_{k}$ denote $x(kh)$, $\lambda (kh)$, $\beta (kh)$, and $b(kh)$, respectively. Rearranging the terms, one obtains for every $k \geq 1$

$$ x_{k+1} + \frac{kh (\beta _{k} + h b_{k}) \nabla \Phi _{\lambda _{k+1}} (x_{k+1})}{\alpha + k} = x_{k} + \frac{k (x_{k} - x_{k-1})}{\alpha + k} + \frac{kh \beta _{k} \nabla \Phi _{\lambda _{k}} (x_{k})}{\alpha + k} $$

or, equivalently,

$$ (\forall k \geq 1) \quad \textstyle\begin{cases} y_{k} := x_{k} + \frac{k}{\alpha + k}(x_{k} - x_{k-1}) + \frac{kh \beta _{k}}{\alpha + k} \nabla \Phi _{\lambda _{k}} (x_{k}), \\ x_{k+1} := \operatorname{prox}_{\frac{kh (\beta _{k} + h b_{k})}{\alpha + k} \Phi _{\lambda _{k}}}(y_{k}). \end{cases} $$

Relation (6), namely,

$$ \nabla \Phi _{\lambda} (x) = \frac{1}{\lambda} \bigl( x - \operatorname{prox}_{ \lambda \Phi} (x)\bigr) \quad \forall x \in H, $$

and the property of the proximal mapping (see, for instance, [16])

$$ \operatorname{prox}_{\mu \Phi _{\lambda}}(x) = \frac{\lambda}{\lambda + \mu} x + \frac{\mu}{\lambda + \mu} \operatorname{prox}_{(\lambda + \mu )\Phi}(x) \quad \forall x \in H, \forall \mu , \lambda > 0, $$

lead to the following formulation of the implicit numerical algorithm

$$ (\forall k \geq 1) \quad \textstyle\begin{cases} y_{k} := x_{k} + \frac{k}{\alpha + k} (x_{k} - x_{k-1}) + \frac{kh \beta _{k}}{\lambda _{k} (\alpha + k)} (x_{k} - \operatorname{prox}_{\lambda _{k} \Phi}(x_{k})), \\ x_{k+1} := \frac{\lambda _{k} (\alpha + k)}{\lambda _{k} (\alpha + k) + kh (\beta _{k} + h b_{k})} y_{k} + \frac{kh (\beta _{k} + h b_{k})}{\lambda _{k} (\alpha + k) + kh (\beta _{k} + h b_{k})} \operatorname{prox}_{ \frac{\lambda _{k} (\alpha + k) + kh (\beta _{k} + h b_{k})}{\alpha + k} \Phi}(y_{k}), \end{cases} $$

where $x_{0}, x_{1} \in H$ are given starting points.

## Polynomial choices for the system parameter functions

According to the previous two sections, to guarantee both the fast convergence rates in Theorem 4 and the convergence of the trajectory to a minimizer of Φ in Theorem 5, also by taking into account Remark 1, it is enough to make the following assumptions on the system parameter functions

(I) $\alpha >1$, and there exists $\varepsilon \in (0, \alpha - 1) $ such that $(\alpha - 3) w(t) - t \dot{w}(t) \geq \varepsilon b(t) $ for every $t \geq t_{0}$;
(II) *β* and *λ* are nondecreasing on $[t_{0}, +\infty )$;
(III) $b(t) > \dot{\beta}(t) + \frac{\beta (t)}{t} $ for every $t \geq t_{0}$;
(IV) $\int _{t_{0}}^{+\infty} [ \frac{\beta ^{2}(t) (\dot{\lambda}(t))^{2} t^{3}}{\lambda ^{4}(t)} - \frac{\dot{\lambda}(t) t^{2} b(t)}{2 \lambda ^{2}(t)} ]_{+} \,dt < +\infty $;
(V) there exists $C > 0 $ such that $\frac{d}{dt} ( t^{2} b(t) ) \leq C t b(t) $ for every $t \geq t_{0}$;
(VI) $\sup_{t \geq t_{0}} \frac{\beta (t)}{t w(t)} < +\infty $;
(VII) $\sup_{t \geq t_{0}} \frac{\lambda (t)}{t} < +\infty $.

In this section, we will investigate the fulfillment of these conditions for

$$ b(t) = b t^{n}, \qquad \beta (t) = \beta t^{m} \quad \text{and} \quad \lambda (t) = \lambda t^{l},$$

where $n,m,l \in \mathbb{R}$, $b, \lambda >0$ and $\beta \geq 0$.

For this choice of *b*, condition (V) is fulfilled.

First, we assume that $\beta =0$. Then the conditions (III), (IV), and (VI) are fulfilled, while the conditions (II) and (VI) are nothing else than $0 \leq l \leq 1$. Condition (I) asks for $\alpha >1$ and for the existence of $\varepsilon \in (0, \alpha - 1)$ such that for every $t \geq t_{0}$

$$ (\alpha - 3 - n - \varepsilon )bt^{n} \geq 0$$

or, equivalently, $\alpha - 3 - n \geq \varepsilon $. To this end, it is enough to have that $\alpha -3 >n$.

In case $\beta >0$, conditions (II) and (VII) are nothing else than $m \geq 0$ and $0 \leq l \leq 1$. Condition (III) reads for every $t \geq t_{0}$

$$ b t^{n} > m \beta t^{m-1} + \beta t^{m-1} = (m + 1) \beta t^{m-1}, $$

or, equivalently,

$$ t^{n - m + 1} > \frac{(m + 1) \beta}{b}. $$

From here, we get

$$ 0 \leq m \leq n + 1, $$

and $b > (m + 1) \beta t_{0}^{m - 1 - n}$.

Condition (VI) requires that

$$ \sup_{t \geq t_{0} } \frac{\beta t^{m}}{t (b t^{n} - \beta m t^{m-1} - \beta t^{m-1})} = \sup_{t \geq t_{0}} \frac{\beta t^{m}}{b t^{n+1} - \beta t^{m} (m + 1)} < +\infty , $$

and it is obviously fulfilled.

Condition (I) asks for $\alpha >1$ and for the existence of $\varepsilon \in (0, \alpha - 1)$ such that for every $t \geq t_{0}$

$$ (\alpha - 3) \bigl(b t^{n} - \beta m t^{m-1} - \beta t^{m-1}\bigr) - t \bigl(b n t^{n-1} -\beta m (m - 1) t^{m-2} - \beta (m - 1) t^{m-2}\bigr) \geq \varepsilon b t^{n}. $$

After simplification, we obtain that for every $t \geq t_{0}$

$$ (\alpha - 3 - n - \varepsilon )b t^{n} + \beta (m + 1) (m + 2 - \alpha ) t^{m-1} \geq 0 $$

or, equivalently,

$$ (\alpha - 3 - n - \varepsilon )b t^{n - m + 1} \geq \beta (m + 1) ( \alpha - m - 2). $$

On the one hand, we have $m = n + 1$ and $(\alpha - 3 -n) ( 1 - \frac{\beta (n + 2)}{b} ) > \varepsilon $, which requires that $\alpha - 3 - n > 0$. On the other hand, we have $m < n + 1$, which also requires that $\alpha - 3 - n > 0$.

Consequently, we have to assume that

$$ \alpha - 3 > n \quad \text{and} \quad b > \frac{\beta (m + 1) (\alpha - m - 2)}{(\alpha - 3 - n) t_{0}^{n - m + 1}}. $$

In this case, there will be always an $\varepsilon \in (0, \alpha - 1)$ such that $\alpha - 3 - n - \varepsilon > 0$ and

$$ b > \frac{\beta (m + 1) (\alpha - m - 2)}{(\alpha - 3 - n - \varepsilon ) t_{0}^{n - m + 1}} > \frac{\beta (m + 1) (\alpha - m - 2)}{(\alpha - 3 - n) t_{0}^{n - m + 1}}, $$

in other words, which satisfies condition (I).

Finally, let us have a closer look at condition (V). This reads as

$$ \int _{t_{0}}^{+\infty} \biggl[ \frac{\beta ^{2} t^{2m} (l \lambda )^{2} t^{2l - 2} t^{3}}{\lambda ^{4} t^{4l}} - \frac{l \lambda t^{l-1} t^{2} b t^{n}}{2 \lambda ^{2} t^{2l}} \biggr]_{+} \,dt < +\infty $$

or, equivalently,

$$ \int _{t_{0}}^{+\infty} \biggl[ \biggl( \frac{\beta l }{\lambda} \biggr)^{2} t^{2m -2l + 1} - \frac{l b}{2 \lambda} t^{n - l + 1} \biggr]_{+} \,dt = \int _{t_{0}}^{+\infty} \biggl[ \biggl( \frac{\beta l }{\lambda} \biggr)^{2} - \frac{l b}{2 \lambda} t^{n + l - 2m} \biggr]_{+} t^{2m -2l + 1} \,dt < +\infty . $$

1. In case

$$ n + l > 2m, $$

there exists $t_{1} \geq t_{0}$ such that for every $t \geq t_{1}$

$$ \biggl( \frac{\beta l }{\lambda} \biggr)^{2} - \frac{l b}{2 \lambda} t^{n + l - 2m} \leq 0. $$

Therefore, we obtain

$$\begin{aligned} & \int _{t_{0}}^{+\infty} \biggl[ \biggl( \frac{\beta l }{\lambda} \biggr)^{2} - \frac{l b}{2 \lambda} t^{n + l - 2m} \biggr]_{+} t^{2m -2l + 1} \,dt \\ &\quad = \int _{t_{0}}^{t_{1}} \biggl[ \biggl( \frac{\beta l }{\lambda} \biggr)^{2} - \frac{l b}{2 \lambda} t^{n + l - 2m} \biggr]_{+} t^{2m -2l + 1} \,dt + \int _{t_{1}}^{+\infty} \biggl[ \biggl( \frac{\beta l }{\lambda} \biggr)^{2} - \frac{l b}{2 \lambda} t^{n + l - 2m} \biggr]_{+} t^{2m -2l + 1} \,dt \\ &\quad = \int _{t_{0}}^{t_{1}} \biggl[ \biggl( \frac{\beta l }{\lambda} \biggr)^{2} - \frac{l b}{2 \lambda} t^{n + l - 2m} \biggr]_{+} t^{2m -2l + 1} \,dt < +\infty , \end{aligned}$$

thus (V) is fulfilled.

2. In case

$$ n + l < 2m, $$

there exist $\delta >0$ and $t_{2} \geq t_{0}$ such that for all $t \geq t_{2}$

$$ \biggl( \frac{\beta l }{\lambda} \biggr)^{2} - \frac{l b}{2 \lambda} t^{n + l - 2m} > \delta . $$

Taking into account that $2m-2l+1 >n + 1 - l \geq -1$, we have

$$\begin{aligned} & \int _{t_{0}}^{+\infty} \biggl[ \biggl( \frac{\beta l }{\lambda} \biggr)^{2} - \frac{l b}{2 \lambda} t^{n + l - 2m} \biggr]_{+} t^{2m -2l + 1} \,dt \\ &\quad \geq \int _{t_{0}}^{t_{2}} \biggl[ \biggl( \frac{\beta l }{\lambda} \biggr)^{2} - \frac{l b}{2 \lambda} t^{n + l - 2m} \biggr]_{+} t^{2m -2 l + 1} \,dt + \delta \int _{t_{2}}^{+\infty} t^{2m - 2l + 1} \,dt = +\infty , \end{aligned}$$

thus, (V) is not fulfilled.

3. It is only left to consider the case

$$ n + l = 2m.$$

Condition (V) becomes

$$ \int _{t_{0}}^{+\infty} \biggl[ \biggl( \frac{\beta l }{\lambda} \biggr)^{2} - \frac{l b}{2 \lambda} \biggr]_{+} t^{2m - 2l + 1} \,dt < +\infty . $$

If $b \geq \frac{2 \beta ^{2} l }{\lambda}$, then it is fulfilled. Otherwise, since $2m - 2l + 1 = n-l+1 \geq -1$, it is not fulfilled.

Summarising, all convergence statements in Theorem 4 and Theorem 5 hold in the two settings

1. $\alpha > 1$, $\beta = 0$, $\alpha - 3 > n$, $0 \leq l \leq 1$, and $b, \lambda > 0$;
2. $\alpha > 1$, $\beta > 0$, $\alpha - 3 > n$, $0 \leq l \leq 1$, $0 \leq m \leq n + 1$, $b > \frac{(m + 1) (\alpha - m - 2) \beta}{(\alpha - 3 - n)t_{0}^{n - m + 1}}$, $\lambda > 0$, and either $2m < n + l$, or $2m = n + l$ and $b \geq \frac{2l \beta ^{2}}{\lambda}$.

### Remark 3

Theorem 4 is providing for the choices $b(t) = b t^{n}$ and $\lambda (t) = \lambda t^{l}$ the following convergence rates

$$ \Phi \bigl(\operatorname{prox}_{\lambda (t)\Phi}\bigl(x(t)\bigr)\bigr) - \Phi ^{*} = o \biggl( \frac{1}{t^{n+2}} \biggr), \qquad \bigl\Vert \operatorname{prox}_{ \lambda (t)\Phi}\bigl(x(t)\bigr) - x(t) \bigr\Vert = o \biggl( \frac{1}{t^{\frac{n}{2} + 1 - \frac{l}{2}}} \biggr) $$

and

$$ \bigl\Vert \nabla \Phi _{\lambda (t)} \bigl(x(t)\bigr) \bigr\Vert = o \biggl( \frac{1}{t^{\frac{n}{2} + 1 + \frac{l}{2}}} \biggr), $$

as $t \to +\infty $. Clearly, the bigger the *n* is, the faster the convergence is. On the other hand, concerning the exponent *l*, things are a bit more complicated: we may gain in one case but inevitably lose in the other. An interesting case is when $l = 0$, which corresponds to *λ* being a constant function. In this case, one can notice a balance between accelerating the convergence of $\| \nabla \Phi _{\lambda (t)} (x(t)) \|$ and slowing the latter for $\|\operatorname{prox}_{\lambda (t)\Phi}(x(t)) - x(t) \|$, since none of them are affected by *l* anymore.

## Numerical experiments

In this section, we will conduct series of experiments to investigate the influence of the system parameters *λ*, *β*, and *b* on the convergence behavior of dynamical system. We will successively fix two of them and vary the last one for this. For the numerical experiments, we will restrict ourselves to the polynomial choices addressed in the previous section $\lambda (t) = t^{l}$, $\beta (t) = t^{m}$, $b(t) = bt^{n}$ with $b = \frac{(m + 1) (\alpha - m - 2) \beta}{(\alpha - 3 - n) t_{0}^{n - m + 1}} + 1$, as well as $x(t_{0}) = x_{0} = 10$, $\dot{x}(t_{0}) = 0$, and $t_{0} = 1$.

### The influence of *b* on the dynamical behaviour

First, let us choose as objective function $\Phi : \mathbb{R} \rightarrow \mathbb{R}_{+}$, $\Phi (x) = |x|$, fix $m = 0$, $\alpha = 9$ and $l = 1$, and vary *n*.

**Figure 1:**
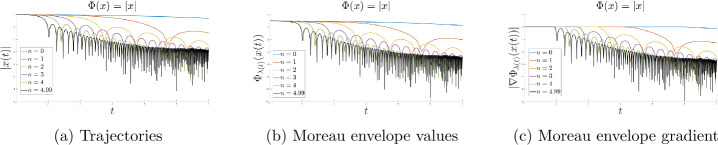
$m = 0$, $\alpha = 9$ and $l = 1$

In Fig. 1, we clearly see that the faster the exponent of the function *b* grows the faster the convergence of the function values of the Moreau envelope and its gradient are, starting with the slowest pace for $n = 0$ and accelerating until $n = 4.99$, confirming the theoretical convergence rates. In addition, the increase in the exponent of *b* also seems to improve the convergence behavior of the trajectory. Fast growing exponents for *b* will improve the convergence greatly; however, as seen in the previous section, they are limited by the upper bound value $\alpha -3$.

### The influence of *λ* on the dynamical behaviour

For the same objective function as in the previous subsection, we study the behavior of the dynamics when varying the exponent *l* to investigate the influence of the function *λ*. To this end, we fix $m = 0$, $\alpha = 9$ and $n = 5 < \alpha -3$, and take for *l* three different values from 0 to 1. We also choose the starting point $x_{0}=1$ to provide a better illustration.

**Figure 2:**
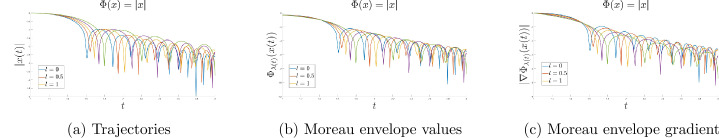
$m = 0$, $\alpha = 9$ and $n = 5$

One can notice in Fig. 2 that the convergence behavior of the function values of the Moreau envelope and its gradient is better, the higher *l* is, whereas, interestingly enough, for the convergence of the trajectories, an opposite phenomenon takes place.

### The influence of *β* on the dynamical behaviour

**Figure 3:**
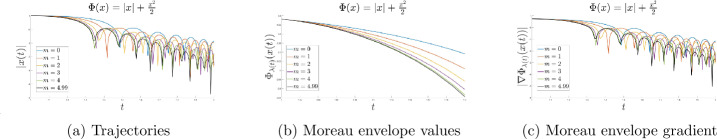
$n = 9$, $\alpha = 13$ and $l = 1$

Let $\Phi : \mathbb{R} \rightarrow \mathbb{R}_{+}$, $\Phi (x) = |x| + \frac{x^{2}}{2}$, $\alpha = 13$, $n = 9 < \alpha -3$ and $l=1$. We vary the exponent *m* such that $2m < n+l$ to study the influence of the function *β* on the convergence behavior of the system. In Fig. 3, we see that, even though *m* does not explicitly appear in the theoretical convergence rates for the gradient of the Moreau envelope and the trajectory of the system, it influences the convergence behavior of both of them as well as of the function values of the Moreau envelope, in the sense that these are faster, the higher the values of *m* are.

**Figure 4:**
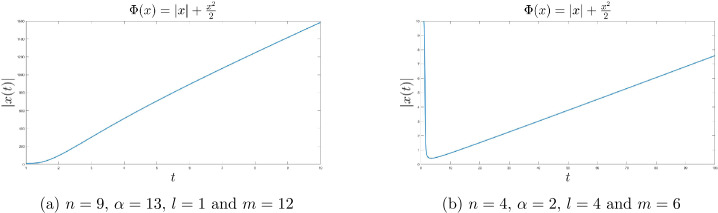
Divergence of the trajectories

Finally, we consider two parameter choices, which lie outside the convergence setting derived in the previous section, and notice that these fundamentally affect the convergence of the trajectory. In Fig. 4(a), we choose *m* such that the condition $2m < n+l$ is violated, and in Fig. 4(b), we choose *α* and *n* such that the condition $\alpha - 3 > n$ is also violated. One can see that in both settings, the trajectories diverge.

## Data Availability

Not applicable.

## Appendix

In this appendix, we collect some lemmas that play an important role in the proof of the main results of the paper. For the proof of the following lemma, we refer to [2].

### Lemma 6

*Suppose that* $f: [t_{0}, +\infty ) \to \mathbb{R}$ *is locally absolutely continuous and bounded from below and there exists* $g \in L^{1}([t_{0}, +\infty ), \mathbb{R})$ *such that for almost all* $t \geq t_{0}$

$$ \frac{d}{dt} f(t) \leq g(t). $$

*Then*, *there exists* $\lim_{t \to +\infty} f(t) \in \mathbb{R}$.

For the proof of the following lemma, we refer to [15].

### Lemma 7

*Let* *H* *be a real Hilbert space and* $x : [t_{0}, +\infty ) \longrightarrow \mathbb{H}$ *a continuously differentiable function satisfying* $x(t) + \frac{t}{\alpha} \dot{x}(t) \to L $ *as* $t \to +\infty $, *with* $\alpha > 0 $ *and* $L \in \mathbb{H} $. *Then* $x(t) \to L $ *as* $t \to +\infty $.

Finally, we state a continuous version of Opial’s Lemma (see [22]), which is used in the proof of the convergence of the trajectory.

### Lemma 8

*Let* *S* *be a non*-*empty subset of a real Hilbert space* *H* *and* $x: [0, +\infty ) \mapsto H$ *a given map*. *Assume that*

- *for every* $z \in S$, $\lim_{t \to +\infty} \| x(t) - z \|$ *exists*;
- *every weak sequential cluster point of the map* *x* *belongs to* *S*.

*Then*, $x(t)$ *converges weakly to some element of* *S* *as* $t \to +\infty $.

## References

[1] Alvarez F., Attouch H., Bolte J., Redont P. (2002). A second-order gradient-like dissipative dynamical system with Hessian-driven damping, application to optimization and mechanics. J. Math. Pures Appl..

[2] Attouch H., Abbas B., Svaiter B.F. (2014). Newton-like dynamics and forward–backward methods for structured monotone inclusions in Hilbert spaces. J. Optim. Theory Appl..

[3] Attouch H., Alvarez F. (2000). The heavy ball with friction dynamical system for convex constrained minimization problems. Optimization.

[4] Attouch H., Alvarez F. (2001). An inertial proximal method for maximal monotone operators via discretization of a nonlinear oscillator with damping. Set-Valued Anal..

[5] Attouch H., Balhag A., Chbani Z., Riahi H. (2022). Fast convex optimization via inertial dynamics combining viscous and Hessian-driven damping with time rescaling. Evol. Equ. Control Theory.

[6] Attouch H., Cabot A. (2018). Convergence of damped inertial dynamics governed by regularized maximally monotone operators. J. Differ. Equ..

[7] Attouch H., Chbani Z., Fadili J., Riahi H. (2022). First-order optimization algorithms via inertial systems with Hessian driven damping. Math. Program..

[8] Attouch H., Chbani Z., Fadili J., Riahi H. (2022). Convergence of iterates for first-order optimization algorithms with inertia and Hessian driven damping. Optimization.

[9] Attouch H., Chbani Z., Peypouquet J., Redont P. (2018). Fast convergence of inertial dynamics and algorithms with asymptotic vanishing viscosity. Math. Program..

[10] Attouch H., Chbani Z., Riahi H. (2019). Fast convex optimization via time scaling of damped inertial gradient dynamics. SIAM J. Optim..

[11] Attouch H., Goudou X., Redont P. (2000). The heavy ball with friction method. The continuous dynamical system, global exploration of the local minima of a real-valued function by asymptotical analysis of a dissipative dynamical system. Commun. Contemp. Math..

[12] Attouch H., László S. (2021). Continuous Newton-like inertial dynamics for monotone inclusions. Set-Valued Var. Anal..

[13] Attouch H., Peypouquet J. (2016). The rate of convergence of Nesterov’s accelerated forward-backward method is actually faster than $\frac{1}{k^{2}}$. SIAM J. Optim..

[14] Attouch H., Peypouquet J. (2019). Convergence of inertial dynamics and proximal algorithms governed by maximal monotone operators. Math. Program..

[15] Attouch H., Peypouquet J., Redont P. (2016). Fast convex optimization via inertial dynamics with Hessian driven damping damping. J. Differ. Equ..

[16] Bauschke H.H., Combettes P.L. (2016). Convex Analysis and Monotone Operator Theory in Hilbert Spaces.

[17] Boţ R.I., Csetnek E.R. (2016). Second order forward–backward dynamical systems for monotone inclusion problems. SIAM J. Control Optim..

[18] Cabot A., Engler H., Gadat S. (2009). On the long time behavior of second order differential equations with asymptotically small dissipation and insights. Trans. Am. Math. Soc..

[19] Cabot A., Engler H., Gadat S. (2009). Second order differential equations with asymptotically small dissipation and piecewise flat potentials. Proceedings of the Seventh Mississippi State–UAB Conference on Differential Equations and Computational Simulations.

[20] May R. (2017). Asymptotic for a second-order evolution equation with convex potential and vanishing damping term. Turk. J. Math..

[21] Nesterov Y. (1983). A method for solving the convex programming problem with convergence rate $O ( \frac{1}{k^{2}} )$. Dokl. Akad. Nauk SSSR.

[22] Opial Z. (1967). Weak convergence of the sequence of successive approximations for nonexpansive mappings. Bull. Am. Math. Soc..

[23] Polyak B.T. (1964). Some methods of speeding up the convergence of iterative methods. USSR Comput. Math. Math. Phys..

[24] Su W., Boyd S., Candès E.J. (2016). A differential equation for modeling Nesterov’s accelerated gradient method: theory and insights. J. Mach. Learn. Res..

